# Maturity-onset diabetes of the young type 5 a MULTISYSTEMIC disease: a CASE report of a novel mutation in the HNF1B gene and literature review

**DOI:** 10.1186/s40842-020-00103-6

**Published:** 2020-08-26

**Authors:** Juan Camilo Mateus, Carolina Rivera, Miguel O’Meara, Alex Valenzuela, Fernando Lizcano

**Affiliations:** 1 Endocrinology Fellowship, School of Medicine and Health Sciences, Rosary University – Fundacion Cardio-Infantil IC, Bogotá, Colombia; 2 Department of Genetics, Fundacion Cardio-Infantil IC, Bogotá, Colombia; 3 Department of Endocrinology, Diabetes and Nutrition, Fundacion Cardio-Infantil IC, Bogotá, Colombia; 4 Center of Biomedical Investigation Universidad de La Sabana, CIBUS, Chia, CU 250008 Colombia

**Keywords:** MODY5, *HNF1B*, Renal hypoplasia, Pancreatic atrophy

## Abstract

**Background:**

Diabetes mellitus with autosomal dominant inheritance, such as maturity-onset diabetes of the young (MODY), is a genetic form of diabetes mellitus. MODY is a type of monogenic diabetes mellitus in which multiple genetic variants may cause an alteration to the functioning of beta cells. The three most known forms of MODY are caused by modifications to the *hnf4a*, *gck*, and *hnf1a* genes. However, other MODY variants can cause multiple alterations in the embryonic development of the endoderm. This is the case in patients presenting with MODY5, who have a mutation of the hepatic nuclear factor 1B (*hnf1b)* gene.

**Case presentation:**

We present the clinical case of a 15 year-old patient with a family history of diabetes mellitus and a classical MODY type 5 (MODY5) phenotype involving the pancreas and kidney, with a novel, unreported mutation in the *hnf1b* gene.

**Conclusions:**

MODY5 is characterised by a mutation in the *hnf1b* gene, which plays an important role in the development and function of multiple organs. It should be suspected in patients with unusual diabetes and multisystem involvement unrelated to diabetes.

**Graphical abstract:**

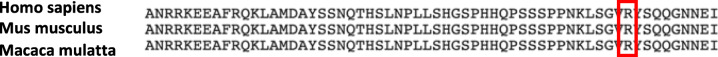

## Background

Diabetes mellitus (DM) with autosomal dominant inheritance, i.e., maturity-onset diabetes of the young (MODY), is a heterogeneous group of diseases caused by gene mutations that result in pancreatic beta-cell dysfunction [1]. Confirmation of a diagnosis of MODY allows for successful patient management, ensuring a healthy pregnancy and the provision of genetic counselling to families [2]. Examination of the relatives of MODY patients makes it possible to diagnose hyperglycaemia in the preclinical phase and allows close monitoring for possible complications derived from this entity.

Typically, the onset of diabetes is during early life, with a mean age of presentation of around 25 years of age [3]. At least 14 different genes have been reported to be involved in the aetiology of MODY [4]. Mutations of the *hnf1a*, *gck* and *hnf4a* genes are the most frequently involved, accounting for 15–25%, 30–50 and 5%, respectively [1]. Mutations in the hepatic nuclear factor 1B (*hnf1b*) gene have been described in patients with MODY type 5 (MODY5), which comprises less than 5% of MODY subtypes [1]. HNF1B is a transcription factor involved in the development of embryonic structures, causing morphological and functional manifestations. It has diverse phenotypes, mainly involving functioning of the endocrine and exocrine pancreas, urogenital tract malformations, renal disease and abnormal liver function.

Here, we report a case of a young patient with an *hnf1b* mutation that has not been previously reported in the literature.

## Case report

A 15-year-old female visited the emergency service because she presented with blurred vision for 1 month, dizziness, fatigue, polydipsia and polyuria, and had lost 4 kg in weight. Upon physical examination, the patient had normal vital signs, with a BMI of 17 kg/m^2^. She was mildly dehydrated, with no other abnormal findings. During admission, blood glucose was above 1000 mg/dl, and glycated haemoglobin (A1C) was 12.8%, with no acute metabolic disorder (no ketosis). Biochemical analysis reported triglycerides of 858 mg/dL, total cholesterol of 255 mg/dL, HDL of 48 mg/dL, aspartate aminotransferase of 26 U/L, alanine aminotransferase of 15 U/L, basal insulin of 8.1 mU/mL and C-peptide of 0.9 ng/mL. Anti-acid decarboxylase (GAD65), anti-insulin, insulinoma-associated protein 2 (IA-2), and anti-zinc transporter 8 (ZNT8) autoantibodies were all negative. A continuous insulin infusion regimen was initially prescribed with subsequent progressive dose reduction due to the normalization of blood glucose control.

She had regular menstrual cycles with a history of left renal hypoplasia, bilateral diminished corticomedullary differentiation and simple renal cysts, with no remarkable changes in the follow-up and conserved normal renal function.

**Fig. 1:**
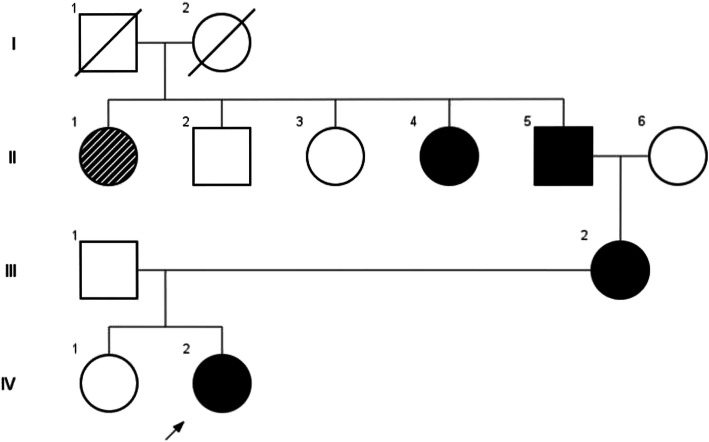
Family pedigree showing individuals with diabetes. People with diabetes are indicated by filled symbols. The parent with a neurocognitive deficit is shown by a circle with oblique lines

Her mother had a history of cystic kidney disease, hypertension, and hypotonic bladder with recurrent urinary tract infections, as well as possible irritable bowel syndrome, but there was no associated hyperglycaemia disorder. Moreover, her mother had 5 pregnancies but only 2 live births; the first one was due to a blighted ovum, the second was a miscarriage and the third was lost due to multiple malformations including a horseshoe, polycystic and duplex kidney. The patient’s grandfather had a history of irritable bowel syndrome and was diagnosed at age 35 with type 2 diabetes due to blood glucose above 700 mg/dl, without ketoacidosis. Furthermore, there was a remarkable history of early diabetes, classified as type 2, in her family, and her great aunt had a moderate neurocognitive deficit (see Fig. 1).

Based on early diabetes development, the absence of type 1 diabetes (T1D) antibodies, the presence of renal dysgenesis and a relevant history of atypical diabetes in her family, MODY5 was suspected and sequencing of the *hnf1b* gene was requested.

The analysis of a trio exome detected the heterozygous variant c.1149delinsTGGCC, p.Arg351Leufs*10 (NM_000458.3) in the *hnf1b* gene. It was identified in the index case and her mother. This variant was classified as being likely pathogenic. It has not been described in this disease, nor is it present within the ClinVar dataset, and is absent from the gnomAD global population dataset.

A protein sequence alignment was performed (NCBI BLAST) evaluating the level of conservation of individual amino acids. The amino acid arginine at position 351 of the HNF1B protein is highly conserved through species, so it is likely to have an important role in the function of the protein (see Table 1). The variant c.1149delinsTGGCC, p.Arg351Leufs*10 in HNF1B creates a shift in the reading frame, which will probably result in the nonsense-mediated decay of the mRNA transcript with functional consequences. A prediction in silico was performed using five bioinformatics tools (MutationTaster, Polyphen2, SIFT, MutPed2, and PMut). All these tools reported that the mutation is pathogenic. The stop codon in the wild type protein is at amino acid residue 558. In contrast, in the mutated protein, the stop codon is at amino acid residue 360, resulting in a truncated protein with an altered function. This mutation locates on the transactivation domain of the HNF1B that is Ser/Pro/Gln-rich, we consider the variant c.1149delinsTGGCC, p.Arg351Leufs*10 in HNF1B to be pathogenic and the cause of MODY5 in the patient.

**Table 1:**
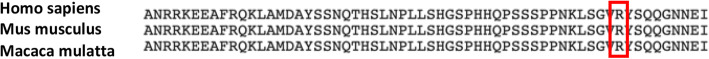
Protein sequence analysis evaluating the level of conservation of the amino acid residue altered by the mutation p.Arg351Leufs*10

**Fig. 2:**
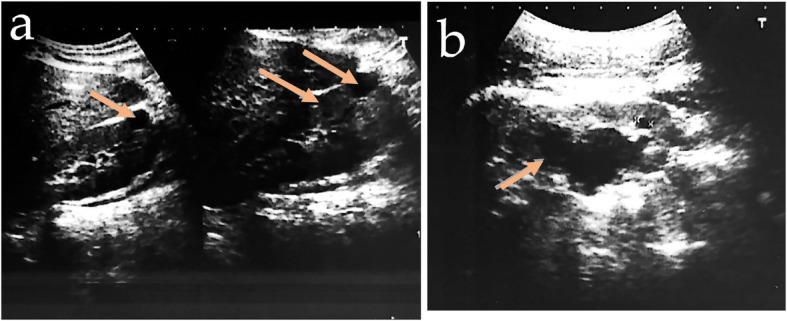
Ultrasonography showing simple renal cysts, diminished corticomedullary differentiation (**a**) and caliectasis (**b**). With the arrow are indicated the different phenotypes changes

After three years of follow-up after initial diagnosis, blood glucose control was found to be within acceptable levels following a diet with carbohydrate counting and frequently performed physical activity. Her weight is 45 kg, with a body mass index of 17.5 kg/m^2^. During follow-up, serum calcium and parathyroid hormone levels were normal, and a low 24-h urine calcium (16.3 mg) was identified. A fasting plasma glucose of 118 mg/dL, A1C of 6.2%, total cholesterol of 221 mg/dL, HDL of 85 mg/dL, triglycerides of 230 mg/dL, uric acid of 6.45 mg/dL, and serum magnesium of 1.28 mg/dL was reported. The liver and renal function was, normal but enalapril was prescribed because of a positive microalbuminuria of 273 mg/g. Faecal elastase was 133 μg/g (reference value > 200 μg/g), so pancreatic enzyme supplements were initiated. Total abdominal ultrasound follow-up showed a reduce pancreas body, a right subcapsular simple renal cyst and left diminished corticomedullary differentiation with caliectasis (see Fig. 2).

## Discussion

In the present case, a heterozygous frameshift mutation variant in the *hnf1b* gene was reported: c.1149delinsTGGCC, p.Arg351Leufs*10; this had not been previously described, but was detected in both the female patient and her mother with a phenotype typical of MODY5. In silico analysis indicated that this mutation is likely to be pathogenic. However, different phenotypes in the family were observed. In the case of the patient, renal and urinary tract malformations were detected, and she also has hypoplasia of the pancreas body associated with early diabetes without beta cell autoantibodies, hypertriglyceridemia and variable hyperglycaemia, with no gastrointestinal symptoms, as well as faecal elastase deficiency. Her mother has a history of pregnancy loss, anatomic and functional urinary tract abnormalities and recurrent urinary tract infections. She was also treated because of early hypertension, which was suspected to be from renal causes. Chronic gastrointestinal symptoms were present, and a probable exocrine pancreas secretion deficit was suspected. No hyperglycaemia was reported, however.

### Epidemiology

Approximately 1–5% of all diabetes cases in the United States and other developed countries are thought to be monogenic [3, 5]. In the United Kingdom, 80% of MODY patients are misdiagnosed as T1D or T2D [6]. Amed et al. [7] reported the incidence rate of MODY in a Canadian population to be 0.4 cases per 100,000 children and youths < 18 years of age. There are 14 MODY subtypes identified, which involve extrapancreatic organs in a small percentage. However, MODY5 frequently compromises renal and other extrapancreatic organs, also known as renal cysts and diabetes syndrome (RCAD syndrome) [8], and accounts for 2–5% of all MODY types [9].

### Genetics

In 1997, Horikawa et al. reported the first case of MODY5 in a Japanese family [10], which was related to a mutation in the *hnf1b* gene. *Hnf1b* contains 9 exons that encode a transcription factor, also known as TCF2, of 557 amino acids; this is a member of the homeodomain-containing superfamily of transcription factors functioning either as homodimers or heterodimers with HNF1A [11]. *Hnf1b* is located on chromosome 17q12, with three functional domains: the transactivation domain, the DNA-binding domain and the dimerisation domain [8, 12]. Heterozygous genetic variations comprise base substitutions leading to missense, nonsense, small deletions or insertions, frameshift and splicing mutations; in some cases, complete gene deletions have been described. Most are grouped in the first four exons of the gene, with exons 2, 4, and the intron 2 splice site being mutation hotspots [8, 13] (refer to supplement Table 1). HNF1B-associated disease results not only from an abnormal DNA binding site but also from the abnormal ability to co-activate proteins, or dimerisation leading to transactivation or transcription disruption [8].

The HNF1B protein plays an important role in the growth of collecting ducts, the renal pelvis and the ureter, and differentiation of the metanephric mesenchyme, which are all key elements for the development of the nephron and collecting system [14]. Also, it regulates the expression of genes such as fibrocystin-1 (*pkhd1*), kinesin-like 12 (*kif12*), suppressor of cytokine signalling 3 (*socs3*) and polycystic kidney disease 2 (*pkd2*), as well as others related to the pathogenesis of the renal cystic disease [14, 15].

Embryogenesis of the pancreas is a dynamic process of gene expression. The pancreas is derived from the foregut of the primitive gut tube [12], which emerges from the endoderm germ layer. Murine models have shown a high expression of *hnf1b* throughout the foregut-midgut region, liver and pancreas buds during the second embryonic week [12]. The consecutive activation of *hnf1b*, hepatocyte nuclear factor 6 (*hnf6*) and pancreatic and duodenal homeobox 1 (*pdx1*) orchestrate the differentiation of endodermal cells into pancreatic progenitors [12]. HNF1B is a key member of the transcriptional factors network (Pdx1, Sox9, Nkx6.1, and Ptf1a) that manage the process of differentiation of pancreatic multipotent cells (PMCs) to endocrine, ductal and acinar cells [16]. HNF1B is required for the proliferation and survival of multipotent cells through modulation of the Fibroblast Growth Factor (FGF) and Notch pathways [12]. Additionally, it regulates expression of the pancreatic islet lineage-defining transcription factor Ngn3 [12] and controls the key cystic disease genes GLIS family zinc finger 3 (*glis3)*, *pkhd1*, mitotic kinesin like2 (*kifl2*), cystin 1 (*cys1*), BicC family RNA binding 1 (*bicc1*) and *hnf6* [12, 17].

### Clinical manifestations

Dubois-Laforgue et al. [18] referred patients with likely MODY characteristics and renal functional and/or morphological anomalies for *hnf1b* gene screening. The prevalence of diabetes was 83% and that of renal malformations was 91%. They found chronic kidney disease (CKD) stages 3 and 4 in 44% of the sample, hypomagnesaemia in 75% and liver test abnormalities in 71%. Diabetes was the first clinical manifestation in 37% of the subjects and renal disease in 39%, while they presented concomitantly both disease in 24%. At diagnosis, 47% of the patients presented symptomatic hyperglycaemia (polyuria, weight loss) but only 5% had ketoacidosis; values of A1C < 7% and ≥ 13% were reported in 32 and 34%, respectively.

HNF1B-associated disease exhibits autosomal dominant inheritance, but de novo mutations account for 50–60% of cases. Additionally, there is no phenotype-genotype correlation; the clinical presentation of the same inherited mutation can even vary significantly within families [11]. The reason for this is not completely understood, but it has been suggested that microenvironment modifiers, stochastic variation in temporal *hnf1b* gene expression and other genes may influence this phenotypic diversity [8, 12].

MODY5 typically develops in adolescence or early adulthood, with a mean age of diagnosis of 24 years, but this can vary widely [8]. Hyperglycaemia is caused by different mechanisms: decreased insulin production due to pancreatic hypoplasia, hepatic insulin resistance and altered glucose-sensing mechanisms [8, 12].

In many cases, pancreatic exocrine deficit has been described with atrophy or a lack of the head and body of the pancreas, with a prevalence of 20–50% [19]. It is usually asymptomatic and documented by reduced faecal elastase [12], as evidenced in our patient. We suspect that symptoms reported as irritable bowel syndrome in the mother and grandfather probably correspond to a deficit in the exocrine pancreas.

Mutations in the *hnf1b* gene are the most frequent cause of monogenic congenital anomalies of the kidney and urinary tract (CAKUT) and remain one of the major causes of CKD in the prenatal and childhood period: 20–31% [20, 21]. However, in a Belgian cohort of 205 CAKUT patients (paediatric and adult), the prevalence of *hnf1b* mutations was 10% [22]. Nagano et al. [9] performed *hnf1b* screening in cases with CAKUT, cystic kidneys, renal dysfunction of unknown cause or Bartter-like syndrome, finding a prevalence of 5.5%. Cystic kidneys were found in 73%, followed by renal hypoplasia (27%). In patients with extra-renal anomalies, 38% developed diabetes, 22% pancreatic malformations, 32% liver abnormalities and 11% female genital malformations [23].

Raaijmakers et al. [22], in a prospective cohort, found that some renal characteristics increased the probability of identifying patients with a mutation in the *hnf1b* gene. The combination of two bilateral renal abnormalities, such as multicystic renal dysplasia, renal agenesis, ectopic kidney, hypoplasia or renal dysplasia, had a relative risk (RR) of 2.9 (95% CI; 1.29–6.67; *P* = 0.010), while cysts of unknown origin had an RR of 6.1 (95% CI; 2.50–15.01; *P* < 0.001) and hypomagnesaemia an RR of 4.2 (95% CI; 1.78, 10.03; *P* = 0.001). They proposed these findings as clinical criteria to restrict genetic analysis and reduce screening costs without missing affected patients.

Additionally, functional kidney anomalies have been reported, such as proteinuria less than 1 g a day, while renal function ranges from normal kidney function in 31.7% to CKD in 55.5% and end stage renal disease (ESRD) in 12.8%. They present as Gitelman-like syndrome (hypomagnesaemia and hypocalciuria) related to the HNF1B transcription function over the FXYD Domain Containing Ion Transport Regulator 2 (*fxyd2*) gene that encodes sodium-potassium ATPase in the distal convoluted tubule, which plays an important role in magnesium reabsorption [14]. Our patient had asymptomatic but slightly low serum magnesium; however, it was believed that she would benefit to continue with a small oral magnesium supplement [24, 25]. Many patients have hyperuricaemia and some present early gout flare, based on regulation of transcription of the uromodulin (*umod)* gene by HNF1B, which is involved in urate transport [14]. Primary hyperparathyroidism has been identified because HNF1B inhibits the transcription of parathormone (*pth)* [26, 27].

Hepatic dysfunction affects 65% of patients with *hnf1b* deletions [19], characterised by elevated serum transaminases, alkaline phosphatase and sometimes mild hyperbilirubinaemia. Histological studies show bile ductopenia, steatosis, and periportal fibrosis, which could lead to cases of neonatal or adult cholestasic hepatopahy. Therefore, some experts have proposed regular laboratory monitoring, annual or biannual abdominal ultrasonography and avoiding hepatotoxic substances [8, 19].

Urogenital tract malformations are seen in up to 50% of patients and it is often seen in female patients associated with fertility problems [19]. Various malformations have been documented, such as a rudimentary or absent uterus, a bicornuate or didelphys uterus, a double vagina or vaginal aplasia. In males, epididymal or seminal vesicle cysts, deferent ducts atresia, hypospadias, varicocele, cryptorchidism, agenesis of the vas deferens, and asthenospermia have been reported in a few cases [4, 26, 28].

**Fig. 3:**
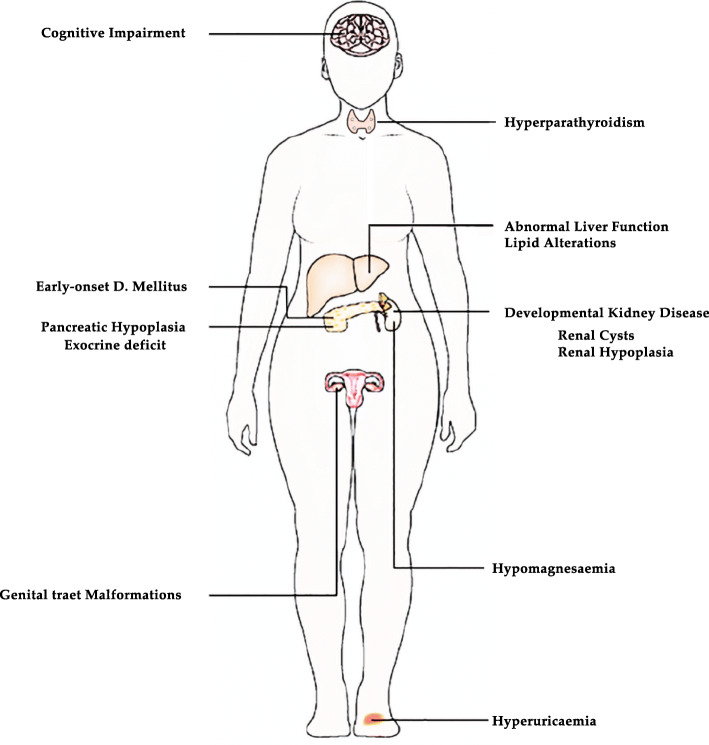
Schematic figure of the main phenotypic abnormalities observed in patients with *hnf1b* gene mutation. In patients with MODY5 the most frequent alteration observed is pancreatic hypoplasia

Neurologic abnormalities, including developmental delay and neuropsychiatric disorders, are characteristic in patients with 17q12 deletion (58 and 27%, respectively) [19]. The *hnf1b* deletion typically also includes the LIM homobox (*lhx1*) and Acetyl-CoA carboxylase alpha (*acaca*) genes. In early brain development, *lhx1* is expressed and plays a role in the differentiation of neural cells and the transcriptional control of axonal guidance. *Acaca* encodes acetyl-CoA carboxylase alpha, a key enzyme in fatty acid metabolism. These two genes have been associated with neurodegenerative diseases, epilepsy, autism, mental retardation and neurodegenerative diseases [29–31]; however, the responsible mechanisms are under investigation (Fig. 3).

### Complications associated with MODY5

Chronic complications associated with MODY5 are not frequently reported in the literature. In a cohort of 27 adult carriers of a *hnf1b* mutation with a median age of 35 years, none developed diabetic retinopathy or neuropathy in the follow-up [32]. On the other hand, Dubois-Laforgue et al. [18] observed retinopathy in 27% of the cohort and peripheral neuropathy in 26% during follow-up; these were associated with older age and a longer duration of diabetes, higher A1C, CKD3–4/ESRD and more frequent insulin therapy. Microalbuminuria was present in 32% of the patients and proteinuria in 26%. Overt coronary artery disease was present in 10% of the subjects and 66 and 31% of patients evolved to CKD 3–4 and ESRD, respectively. In a Japanese cohort [9], the kidney disease progressed to CKD 4 or 5 in 13.7%, with 3% requiring renal transplantation.

Diabetes might present as new-onset diabetes after transplantation (NODAT); prior analysis of the *hnf1b* gene should be considered in all individuals with unexplained congenital anomalies of the kidneys and urinary tract undergoing renal transplantation to improve post-transplant management [8, 12].

Faguer et al. [33] developed a 17-item *hnf1b* risk score as a screening tool. A score of more than 8 points is suggestive of MODY5 disease (sensitivity 98%, specificity 41.1%, PPV 19.8%, NPV 99.4%). However, this score lacks external validation and is reported to lead to false negatives [9, 26].

### Treatment

The current treatments have not been completely standardised because of the small number of cases and lack of large cohorts or randomised clinical trials. The hyperglycaemic management of MODY5 should begin with a stringent diet created by a nutritionist. Although some cases can be managed (or initially) with dietary recommendations solely [18, 34], as is the case reported in this paper, metformin has been tested in all MODY patients. Its efficacy in MODY5 is significantly lower than that of sulphonylureas; therefore, metformin is not routinely recommended [35]. There are some interesting data for MODY3 treatment with incretins, but this is usually added to insulin or sulphonylureas management. We did not find experience with incretin therapy in MODY5. Glinides or sulphonylureas have been an option for treatment with variable glycaemic control and a response period [4, 18, 28, 35]; however, it has been reported that some MODY5 patients do not respond adequately to sulfonylureas [4] as a consequence of hypoplasia and pancreatic dysfunction. The majority of patients require insulin treatment during the follow-up [36]. In a cohort of patients initially treated with diet or oral anti-diabetic agents, insulin was initiated in 67% of cases. The median insulin dosage was 0.55 IU/kg/day (IQR 0.39–0.70). Surprisingly, the same cohort exhibited residual insulin secretion in 80% of cases [18]. Although our patient has not required insulin in recent years, we have shown a slow increase in blood glucose recently, but with no anti-diabetic drug indication at present. She will probably require anti-hyperglycaemic treatment in the next few years, as reported for other patients. Our group suspects that some patients secrete insulin with adequate biological activity based on affected organogenesis pathways and pancreatic insulin synthesis, and that their hepatic insulin sensitivity is sufficient to avoid metabolic disorders.

Proteinuria has to be treated with anti-proteinuric agents as a recommended treatment [37]. Pancreatic exocrine insufficiency is treated with pancreatic enzyme supplements. Gout can be prevented and treated by usual medical care and nutritional support.

## Conclusions

In conclusion, we described a case of MODY5 with an unpublished mutation (c.1149delinsTGGCC, p.Arg351Leufs) in the *hnf1b* gene. MODY5 has a wide clinical presentation, thus, it should not be considered just a glucose metabolic disease, but rather a multi-systemic entity with a broad phenotype spectrum that requires an integral and complete follow-up.

## The view of Ms. G.P.

Having diabetes MODY type 5 lead me to feel frustration and hope. Finding my diagnosis took more than 18 years. When the endocrinologist told me I was diabetic, I asked myself why me? When the insulin started to lower my glucose levels, I had a moment of hope, as I could eat normally again, without having to think about what I could and could not eat. However, the frustration returned when my glucose levels started slightly rising, but it was not enough to go back to insulin. Now, I have a strict diet that I have to follow, and I feel frustration every time I cannot eat something.

However, I also feel hope, as I know that everything happens for a reason, and this happened to me so that I could share my story, and so that my case could help to spread awareness of my condition and continue to motivate research for a treatment or cure. If not for this, then for a more holistic approach for patients.

## Supplementary information

**Additional file 1.**

## Data Availability

The datasets used and/or analysed during the current study are available from the corresponding author on reasonable request.

## Abbreviations

DM: Diabetes mellitus
MODY: Maturity-onset diabetes of the young
HNF1B: Hepatic nuclear factor 1B
MODY5: Maturity-onset diabetes of the young type 5
A1C: Glycated haemoglobin
GAD65: Acid decarboxylase
IA-2: Insulinoma-associated protein 2
ZNT8: Islet zinc transporter 8
TCF2: Transcription factor 2
GLUT2: Glucose transporter type 2
HDL: High density lipoprotein
DNA: Deoxyribonucleic acid
RCAD: Renal cysts and diabetes syndrome
CAKUT: Congenital anomalies of the kidney and urinary tract
CKD: Chronic kidney disease
ESRD: End stage renal disease
T1D: Type 1 diabetes
T2D: Type 2 Diabetes
NODAT: New-onset diabetes mellitus after transplantation
PMCs: Pancreatic multipotent cells
FGF: Fibroblast Growth Factor
RR: Relative risk
