# Supplementary material for: Maturity-onset diabetes of the young type 5 a MULTISYSTEMIC disease: a CASE report of a novel mutation in the HNF1B gene and literature review
Source: Clin Diabetes Endocrinol. 2020 Aug 26;6:16. doi: 10.1186/s40842-020-00103-6 (PMC7448977; doi:10.1186/s40842-020-00103-6)
Supplement: Supplementary file 1 — Additional file 1. [file 40842_2020_103_MOESM1_ESM.docx]

Table 1. More frequent mutations of the *hnf1b* gene in patients suffering 1 MODY 5. (supplement 1)

| **Mutation ^a^** | **Codon** | **Predicted Effect ^b^** | **Clinical feature** | **Characteristics *** | **Reference** |
| --- | --- | --- | --- | --- | --- |
| Exon 1 |  |  |  |  |  |
| c.3G>A | 1 | ms, p.Met1Ile | Structural renal  malformations | Bilateral cortical microcysts; father diabetes and renal cysts | (1) |
| c.18delG | 6 | fs, p.Ser7Argfs*7 | Functional renal and parathyroid dysfunction | ESRD, hyperparathyroidism | (2) |
| c.46delC | 16 | fs, p.Leu16 | Structural and functional renal  anomalies | Bilateral hypoplasia and hyperechogenicity kidneys, CKD | (3) |
| c.207_211delCGCCA | 69-71 | fs, p.Lys71Alafs*52 | Structural renal and urinary tract  anomalies  Structural heart anomalies  Endocrine pancreas insufficiency | Bilateral MCD with reducing size renal, pelvis ectasia, pancreatic hypoplasia, bilateral epididymal cysts, diabetes | (4) |
| c.211delAAGGGCC | 71-73 | fs, p.Lys71fs | Structural renal  malformations | Hypodysplastic kidneys  with microcysts, CKD GFR 45 ml/min at 28 years; father hyperuricaemic nephropathy | (1) |
| c.226G>T | 76 | Gly76Cys | Structural and functional renal  anomalies | Bilateral cortical and unilateral medullary renal cysts, MCD. Bilateral hypoplasia and hyperechogenicity kidneys. CKD. | (3,5) |
| c.232G>T | 78 | ns, p.Glu78X | Structural and functional renal anomalies, genital tract malformations | 1. Bilateral cortical cysts, ESRD at 3 months  2. Septated uterus, MCD | (1) |
| c.322delG | 108 | fs, p.Ala108fs | Structural and functional renal anomalies disorders, genital tract malformations.  Endocrine pancreas insufficiency. | Hyperuricaemic  nephropathy, ESRD at 33  years; father hyperuricaemic nephropathy and diabetes | (1) |
| c.329T>G | 110 | ms, p.Val110Gly | Structural renal  malformations | Glomerulocystic kidney disease | (6) |
| Intron 1 |  |  |  |  |  |
| IVS1 c.345-1G>A |  | spl. | Structural and functional renal anomalies | 1. Bilateral hyperechogenic, cortical cysts, neonatal renal failure  2. Unilateral agenesis,  single hyperechogenic  kidney CKD GFR 25 ml/min at 17 years | (1) |
| Exon 2 |  |  |  |  |  |
| c.406C>T | 136 | ns, p.Gln136X | Structural renal  malformations | 1. Bilateral cortical cysts and hyperechogenicity  2. Bilateral cortical and unilateral medullary cysts. Bilateral hyperechogenicity and hypoplasia kidneys | (3,5) |
| c.434T>A | 145 | ms, p.Leu145Gln | Structural and endocrine pancreas insufficiency. | Diabetes, agenesis of the tail and body of the pancreas, unilateral renal cysts; father diabetes | (7) |
| c.452C>G | 151 | ms, p.Ser151Cys | Structural renal  malformations | Unilateral MCD, unilateral cortical cysts | (1) |
| c.476C>T | 159 | ms, p.Pro159Leu | Structural renal  malformations | Bilateral or isolated hyperechogenic kidneys | (1) |
| c.494 G>A | 165 | ms, p.Arg165His | Structural renal  malformations | Small hyperechogenic  kidneys, CKD GFR 16 ml/min at 4 years; father with renal  failure and diabetes | (5,8,9) |
| c.494G>C | 165 | ms, p.Arg165Pro | Structural and functional renal anomalies, structural pancreas  malformations | Bilateral hyperechogenic and hypoplastic kidneys, CKD GFR 32 ml/min at 10 years, pancreatic hypoplasia | (1) |
| c.513G>A | 171 | ns, p.Trp171X | Structural renal  malformations | Bilateral hyperechogenic  kidneys and bilateral cortical cysts | (1) |
| c.526C>T | 176 | ns, p.Gln176X | Structural renal  malformations | Renal cysts | (6) |
| c.534delG | 176 | fs, p.Glu178 | Structural renal  malformations | Bilateral cortical cysts, hyperechogenic kidneys | (3) |
| c.541C>T | 181 | ns, p.Arg181X | Endocrine pancreas insufficiency | Diabetes | (10) |
| c.544C>T | 182 | ns, p.Gln182X | Structural and functional renal  anomalies  Endocrine pancreas insufficiency | 1. Hyperechogenic kidneys; diabetes in the mother  2. Unilateral MCD, another  kidney with cortical cysts  3. Single hypoplastic  hyperechogenic kidney,  CKD GFR 55 ml/min at 7 years | (1,5,8) |
| Intron 2 |  |  |  |  |  |
| IVS2 +1G>C |  | spl. |  | Unilateral cortical and medullary cysts | (3) |
| IVS2 c.544+6delAAGT |  | spl. | Structural, functional renal and urinary tract  anomalies.  Endocrine pancreas insufficiency  Hepatic disorders | 1. Renal cysts and VUR; mother with unilateral cysts and gestational diabetes  2. Single kidney, gout, CKD GFR 25 ml/min at 65 years; mother and maternal cousin with renal failure; daughter with a single kidney  3. Cysts, CKD GFR 60 ml/min at 33 years, diabetes, hyperuricaemia, elevated liver enzymes, hypomagnesaemia; father  with ESRD | (1,6) |
| IVS2 +1G>A |  | spl. |  | 1. Gout, familial juvenile hyperuricaemic nephropathy, CKD, diabetes  2. Gout, familial juvenile hyperuricaemic nephropathy | (6) |
| Exon 3 |  |  |  |  |  |
| c.716A>G | 239 | ms, p.Gly239Glu | Structural, functional pancreas, renal, liver anomalies | Diabetes, atrophy of the pancreas, CKD, hyperuricemia, and liver dysfunction | (11) |
| c.717delG | 239 | fs, p.Ser242fs | Structural renal  malformations  Endocrine pancreas insufficiency | Bilateral hyperechogenic kidneys, cortical microcysts; father with renal cysts  and diabetes | (1) |
| c.715_717del | 239-240 | if, p.Gly239del | Structural, renal and urinary tract  anomalies  Structural heart anomalies  Endocrine pancreas insufficiency | Hyperechogenic kidneys,  bilateral renal cysts, kidney enlargement, pelvic ectasia,  bilateral megaureter, diabetes, atrial septal defect | (12) |
| c.766C>T | 256 | ms, p.Pro256Ser | Structural renal  malformations | Bilateral hyperechogenic kidneys, bilateral cortical cysts | (1) |
| Intron 3 |  |  |  |  |  |
| IVS3 c.809+1G>A |  | sp | Structural, functional renal and urinary tract  anomalies  Endocrine pancreas insufficiency | Bilateral hyperechogenic kidneys, bilateral cortical cysts, unilateral UPJ obstruction, neonatal renal failure; family history of diabetes | (1) |
| Exon 4 |  |  |  |  |  |
| c.840delC | 280 | fs, p.Pro280fs | Structural renal and urinary tract  anomalies.  Structural pancreas  malformations | Enlarged kidneys, large cysts, pelvic ectasia, duplicity, pancreas hypoplasia | (1,13) |
| c.883C>T | 295 | ms, p.Arg295Cys | Structural, functional renal anomalies  Endocrine pancreas insufficiency and parathyroid dysfunction | 1. Small and cystic kidneys; mother with renal cysts and CKD  (precise GFR unknown)  2. Cortical cysts,  hyperuricaemia, neonatal  renal failure  3. Diabetes, hyperparathyroidism | (2,5,14) |
| c.884G>C | 295 | ms, p.Arg295Pro | Structural renal  Malformations  Endocrine pancreas insufficiency  Malignancy | Renal cysts, diabetes and breast cancer | (6) |
| c.895T>G | 299 | ms, p.Trp299Gly | Structural, functional renal anomalies | Bilateral hyperechogenic kidneys, bilateral cortical microcysts, CKD (GFR 51 ml/min at 3 years) | (1) |
| Exon 4 deletion c.810_1045del236 | 270-349 | fs, p.Arg270fs | Structural, functional renal and urinary tract  anomalies. | Unilateral MCD, absence of hypertrophy of the contralateral kidney, VUR, CKD (GFR 65 ml/min at 4 years) | (1,3,15) |
| c.931C>T | 311 | ns, Gln311X | Structural and functional renal  anomalies. | Bilateral cortical and unilateral medullary renal cysts, multicystic dysplasia. Bilateral renal hypoplasia and hyperechogenicity, CKD | (3) |
| c.1046-294_1206 +704del | 349_402 | p.Gly349_Met402del | Functional renal, pancreas and liver  anomalies. | Diabetes, CKD, elevated liver enzyme. | (5) |
| c.1136C>A | 379 | ns, p.Ser379X | Structural, functional renal and genital tract  anomalies.  Structural pancreas  malformations | Hyperechogenic kidneys,  cortical microcysts, CKD (GFR 61 ml/min at 15 years), didelphic uterus, pancreatic hypoplasia | (1) |
| c.1360C>T | 454 | ns, p.Gln454X | Structural renal  malformations | Unilateral MCD, other kidney hyperechogenic with cortical microcysts. | (1) |
| c.1363_1364delAG | 455 | fs, p.Ser455fs | Structural renal  malformations | Bilateral hyperechogenic  and cortical cysts, left  hypoplastic kidney | (1,16) |
| Exon 7 |  |  |  |  |  |
| c.1395C>G | 465 | ms, p.Ser465Arg | Endocrine pancreas insufficiency | 1. Diabetes treated with OHA  2. Diabetes treated with diet | (17) |
| Complete deletions |  |  |  |  |  |
| 17q12del |  |  | Functional central nervous system development anomalies  Behavioural alterations  Structural heart, renal anomalies  Structural renal  Malformations  Functional pancreas, renal, urogenital, liver, parathyroid anomalies | 1. Learning difficulties, oppositional behaviour, ventricle septum defect  2. Bilateral renal cysts, mild intellectual disability, Asperger syndrome, hyperactivity, restlessness, abnormal social behaviour  3. Motor delay in childhood, learning and concentration difficulties, low-normal IQ, autistic-like behaviour, increased hepatic enzymes  4. Bilateral renal cysts, CKD  5. Recurrent urinary tract infections in childhood, bilateral renal cysts, ESRD, motor delay in childhood, increased hepatic enzymes, biliary cirrhosis due to liver cysts  6. Right kidney agenesis, left kidney hypoplasia, recurrent interstitial nephritis, end-stage renal disease, uterus aplasia, ovarian cysts  7. CKD, hypomagnesaemia, hyperparathyroidism, hypophosphatemia  8. Bilateral renal and unilateral medullary cysts, MCD, hypoplasia, hyperechogenicity kidneys. CKD | (2,3,5,12) |

^a^ Mutations are numbered according to the *Hnf1b* cDNA reference sequence (GenBank NM_000458.2), where the nucleotide +1 corresponds to the A of the ATG initiation codon. The mutation numbering and nomenclature was changed whenever necessary to follow standard recommendations

^b^ Predicted effect: fs, frameshift mutation; if, in-frame deletion or insertion; indel, insertion and deletion; ms, missense mutation; ns, nonsense mutation; sp, splice site mutation

* There are various phenotypes described with the same mutation. Each section is split into numbered sections.

MCD: Multicystic kidney disease; VUR: Vesicoureteral reflux; IQ: Intelligence quotient; ESRD: End-stage renal disease, CKD: Chronic kidney disease; OHA: oral hypoglycaemic agents

**Table bibliography**

1. Heidet L, Decramer S, Pawtowski A, Morinière V, Bandin F, Knebelmann B, et al. Spectrum of *HNF1B* Mutations in a Large Cohort of Patients Who Harbor Renal Diseases. Clin J Am Soc Nephrol. 2010 Jun;5(6):1079–90.

2. Ferrè S, Bongers EMHF, Sonneveld R, Cornelissen EAM, van der Vlag J, van Boekel GAJ, et al. Early Development of Hyperparathyroidism Due to Loss of *PTH* Transcriptional Repression in Patients With HNF1β Mutations? J Clin Endocrinol Metab. 2013 Oct;98(10):4089–96.

3. Ulinski T, Lescure S, Beaufils S, Guigonis V, Decramer S, Morin D, et al. Renal Phenotypes Related to Hepatocyte Nuclear Factor-1β ( *TCF2* ) Mutations in a Pediatric Cohort. J Am Soc Nephrol. 2006 Jan;17(2):497–503.

4. Carrillo E, Lomas A, Pinés PJ, Lamas C. Long-lasting response to oral therapy in a young male with monogenic diabetes as part of HNF1B-related disease. Endocrinol Diabetes Metab Case Rep [Internet]. 2017 Jun 23 [cited 2020 Apr 16];2017. Available from: https://edm.bioscientifica.com/view/journals/edm/2017/1/EDM17-0052.xml

5. Bellanne-Chantelot C, Clauin S, Chauveau D, Collin P, Daumont M, Douillard C, et al. Large Genomic Rearrangements in the Hepatocyte Nuclear Factor-1 (TCF2) Gene Are the Most Frequent Cause of Maturity-Onset Diabetes of the Young Type 5. Diabetes. 2005 Nov 1;54(11):3126–32.

6. Edghill EL, Bingham C, Slingerland AS, Minton JAL, Noordam C, Ellard S, et al. Hepatocyte nuclear factor-1 beta mutations cause neonatal diabetes and intrauterine growth retardation: support for a critical role of HNF-1? in human pancreatic development. Diabet Med. 2006 Dec;23(12):1301–6.

7. Kato T, Tanaka D, Muro S, Jambaljav B, Mori E, Yonemitsu S, et al. A Novel p.L145Q Mutation in the HNF1B Gene in a Case of Maturity-onset Diabetes of the Young Type 5 (MODY5). Intern Med. 2018 Jul 15;57(14):2035–9.

8. Bellanné-Chantelot C, Chauveau D, Gautier J-F, Dubois-Laforgue D, Clauin S, Beaufils S, et al. Clinical Spectrum Associated with Hepatocyte Nuclear Factor-1β Mutations. Ann Intern Med. 2004 Apr 6;140(7):510.

9. Wang C, Zhang R, Lu J, Jiang F, Hu C, Zhou J, et al. Phenotypic heterogeneity in Chinese patients with hepatocyte nuclear factor-1β mutations. Diabetes Res Clin Pract. 2012 Jan;95(1):119–24.

10. Horikawa Y, Iwasaki N, Hara M, Furuta H, Hinokio Y, Cockburn BN. Mutation in hepatocyte nuclear factor-1 beta gene (TCF2) associated with MODY. Nat Genet. 1997;17:384–5.

11. Luo Y, Dai Z, Li L, Shan X, Wu C. Hepatocyte nuclear factor 1β maturity-onset diabetes of the young in a Chinese child presenting with hyperglycemic hyperosmolar state. Acta Diabetol. 2017 Oct;54(10):969–73.

12. Vasileiou G, Hoyer J, Thiel CT, Schaefer J, Zapke M, Krumbiegel M, et al. Prenatal diagnosis of *HNF1B* ‐associated renal cysts: Is there a need to differentiate intragenic variants from 17q12 microdeletion syndrome? Prenat Diagn. 2019 Nov;39(12):1136–47.

13. Madariaga L, Morinière V, Jeanpierre C, Bouvier R, Loget P, Martinovic J, et al. Severe Prenatal Renal Anomalies Associated with Mutations in *HNF1B* or *PAX2* Genes. Clin J Am Soc Nephrol. 2013 Jul 3;8(7):1179–87.

14. Bergmann C, von Bothmer J, Ortiz Brüchle N, Venghaus A, Frank V, Fehrenbach H, et al. Mutations in Multiple PKD Genes May Explain Early and Severe Polycystic Kidney Disease. J Am Soc Nephrol. 2011 Nov;22(11):2047–56.

15. Decramer S, Parant O, Beaufils S, Clauin S, Guillou C, Kessler S, et al. Anomalies of the *TCF2* Gene Are the Main Cause of Fetal Bilateral Hyperechogenic Kidneys. J Am Soc Nephrol. 2007 Mar;18(3):923–33.

16. Barbacci E, Chalkiadaki A, Masdeu C, Haumaitre C, Lokmane L, Loirat C, et al. HNF1 β/TCF2 mutations impair transactivation potential through altered co-regulator recruitment. Hum Mol Genet. 2004 Dec 15;13(24):3139–49.

17. Furuta H. Nonsense and Missense Mutations in the Human Hepatocyte Nuclear Factor-1 Gene (TCF2) and Their Relation to Type 2 Diabetes in Japanese. J Clin Endocrinol Metab. 2002 Aug 1;87(8):3859–63.
